# Association between dietary nitrate and nitrite intake and site-specific cancer risk: evidence from observational studies

**DOI:** 10.18632/oncotarget.10917

**Published:** 2016-07-29

**Authors:** Li Xie, Miao Mo, Hui-Xun Jia, Fei Liang, Jing Yuan, Ji Zhu

**Affiliations:** ^1^ Clinical Statistics Center, Fudan University Shanghai Cancer Center, Shanghai, China; ^2^ Department of Radiation Oncology, Fudan University Shanghai Cancer Center, China; ^3^ Department of Oncology, Shanghai Medical College, Fudan University, Shanghai, China

**Keywords:** nitrate, nitrite, cancer, risk, meta-analysis

## Abstract

Epidemiological studies have reported inconsistent findings on the association between dietary nitrate and nitrite intake and cancer risk. We performed a meta-analysis of epidemiological studies to summarize available evidence on the association between dietary nitrate and nitrite intake and cancer risk from published prospective and case-control studies. PubMed database was searched to identify eligible publications through April 30^th^, 2016. Study-specific relative risks (RRs) with corresponding 95% confidence interval (CI) from individual studies were pooled by using random- or fixed- model, and heterogeneity and publication bias analyses were conducted.

Data from 62 observational studies, 49 studies for nitrates and 51 studies for nitrites, including a total of 60,627 cancer cases were analyzed. Comparing the highest vs. lowest levels, dietary nitrate intake was inversely associated with gastric cancer risk (RR = 0.78; 95%CI = 0.67-0.91) with moderate heterogeneity (*I^2^* = 42.3%). In contrast, dietary nitrite intake was positively associated with adult glioma and thyroid cancer risk with pooled RR of 1.21 (95%CI = 1.03-1.42) and 1.52 (95%CI = 1.12-2.05), respectively. No significant associations were found between dietary nitrate/nitrite and cancers of the breast, bladder, colorectal, esophagus, renal cell, non-Hodgkin lymphoma, ovarian, and pancreas. The present meta-analysis provided modest evidence that positive associations of dietary nitrate and negative associations of dietary nitrite with certain cancers.

## INTRODUCTION

Nitrate and nitrite from food and water are precursors of endogenously formed N-nitroso compounds (NOCs). Results from animal studies and mechanisms describing DNA damage suggest that these compounds are carcinogenic in humans [1, 2]. Ingested nitrate is reduced to nitrite by the bacterial flora in the mouth and digestive tract. In turn, nitrite reacts with amines, amides and other nitrosation precursors in the gastrointestinal tract to form NOCs. Endogenous nitrosation is estimated to account for 45-75% of total NOCs exposure [3]. Acceptable daily intake values have been set for baby foods because high exposure of nitrate would cause methemoglobinemia in infants [4]. However, the regulatory limits for nitrate/nitrite in food have not been extensively studied in relation to other health outcomes. Dietary intake of nitrate and nitrite may be an important cancer risk factor but the research continues to be monitored.

Literature of dietary nitrate/nitrite and cancer risk has been growing but results have been inconsistent. The discrepancy might be partly due to differences in study populations and design, and partly due to the insufficient statistical power of individual studies. Therefore, we conducted a meta-analysis of all observational studies published between the dates of database inception and April 30^th^, 2016 to summarize available evidence on the association between both dietary nitrate and nitrite intake and cancer risk.

## RESULTS

### Literature search

**Figure 1:**
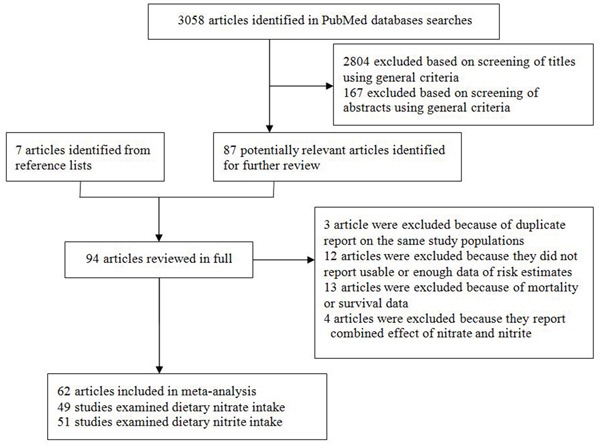
Selection of studies for inclusion in the meta-analysis

Figure 1 illustrates the flow diagram of the literature search and study selection. We identified 3058 potentially relevant articles from search of PubMed databases. Of these, 2804 and 167 articles were excluded based on titles and abstracts using general criteria respectively, leaving 87 articles for full-text review. Seven articles were identified by hand searching of the references list of these articles. A total of 94 articles went on full-text review. During this review, 3 articles was excluded because of duplicate reports from the same study population, 12 articles were excluded because they did not report usable or enough data of risk estimates and 13 articles were excluded because of mortality or survival data, 4 articles were excluded because they report combined association of nitrate and nitrite. The remaining 62 articles were included in this meta-analysis.

### Characteristics of the studies

Characteristics of the 62 included articles are shown in Table 1. The included articles, which reported the combined 60,627 cases and 4,730,572 non-cases, were published between 1985 and 2016 and consist of 24 prospective studies [5–28] and 38 case-control studies [29–66].

**Table 1:** Characteristics of studies included in the meta-analysis^§^

Cancer site	No. of studies	Study design		Area				Sex^*^			Adjusted estimates^†^
		Cohort	C–C	Europe	North America	Asia	Others/mixed	M	F	M+F	
***Nitrate***											
Adult glioma	5	2	3	1	3	-	1	1	1	4	2
Bladder	6	3	3	1	5	-	-	1	1	4	3
Breast	3	2	1	-	2	1	-	-	3	-	2
Colorectal	6	4	2	1	4	1	-	-	2	4	6
Endometrial	1	-	1	-	1	-	-	-	1	-	1
Esophageal	4	2	2	1	3	-	-	1	1	3	4
Gastric	15	6	9	8	5	1	1	1	1	14	8
Head and neck	1	1	-	1	-	-	-	-	-	1	1
Hepatocellular carcinoma	1	1	-	-	1	-	-	-	-	1	1
Larynx	1	-	1	-	1	-	-	-	-	1	1
Leukemia	1	1	-	-	1	-	-	-	-	1	1
Lung and bronchus	1	1	-	-	1	-	-	-	-	1	1
Non-Hodgkin lymphoma	4	1	3	-	4	-	-	-	2	2	4
Ovarian	3	3	-	-	3	-	-	-	3	-	3
Oral cavity	1	-	1	-	1	-	-	-	-	1	1
Pancreatic	3	2	1	-	3	-	-	1	2	1	1
Prostate	1	1	-	-	1	-	-	1	-	-	1
Renal cell	3	2	1	-	3	-	-	-	1	2	3
Skin (Melanoma)	1	1	-	-	1	-	-	-	-	1	1
Thyroid	3	3	-	-	2	1	-	-	2	1	3
Uterine corpus	1	1	-	-	1	-	-	-	-	1	1
***Nitrite***											
Adult glioma	6	2	4	1	4	-	1	1	1	5	3
Bladder	4	1	3	-	4	1	-	2	2	2	2
Breast	4	3	1	1	3	-	-	1	3	-	4
Colorectal	6	4	2	2	3	1	-	-	1	5	5
Esophageal	7	3	4	2	5	-	-	1	1	6	7
Gastric	19	5	14	10	6	-	3	1	1	18	13
Head and neck	1	1	-	1	-	-	-	-	-	1	1
Hepatocellular carcinoma	1	1	-	-	1	-	-	-	-	1	1
Larynx	1	1	-	-	1	-	-	-	-	1	1
Lung and bronchus	1	1	-	1	-	-	-	-	-	1	1
Nasopharyngeal	1	1	-	-	-	1	-	-	-	1	-
Non-Hodgkin lymphoma	4	-	4	-	4	-	-	1	-	3	3
Ovarian	3	3	-	1	2	-	-	-	3	-	3
Oral cavity	1	1	-	-	1	-	-	-	-	1	1
Pancreatic	2	1	1	-	2	-	-	1	1	1	2
Prostate	2	2	-	1	1	-	-	2	-	-	2
Renal cell	2	1	1	-	2	-	-	-	-	2	2
Thyroid	2	2	-	-	1	1	-	-	1	1	2

* Studies that reported gender-specific estimates were counted twice (in both M and W columns).

† The site-specific list of main confounders considered is total energy intake.

§ C-C=Case-control, M=Male, F=Female.

Among the included prospective studies, one article consisted of 3 cohorts. Therefore, a total of 24 prospective studies included 26 cohorts. Of the 24 prospective studies, 17 studies were conducted in the United States, 4 studies in Europe, 2 studies in China, and one study was conducted in United Kingdom. Cohort sizes ranged from 9,985 to 545,770, and the number of cancer cases varied from 45 to 9,305 cases.

Of 38 case-control studies, 21 studies were conducted in the United States, 7 studies in Europe, 3 studies were conducted in Asia, 2 studies each were conducted in Canada, Mexico, and Uruguay, and one study was conducted in Australia, The number of cases enrolled in these studies ranged from 79 to 1760 cases, and the number of control subjects varied from 128 to 2481 subjects. Control subjects were drawn from the general population in 27 studies, hospitals in 11 studies.

### Dietary nitrate intake and site-specific cancer risk

Twenty-two prospective and twenty-eight case-control studies investigated the association between dietary nitrate intake and cancer risk. Among the included studies, 15 studies were on gastric cancer, 6 studies each on colorectal cancer and non-Hodgkin lymphoma, 5 studies each on adult glioma and bladder cancer, 4 studies on esophageal cancer, 3 studies each on breast cancer, pancreatic cancer, renal cell cancer and thyroid cancer and ovarian cancer.

**Figure 2:**
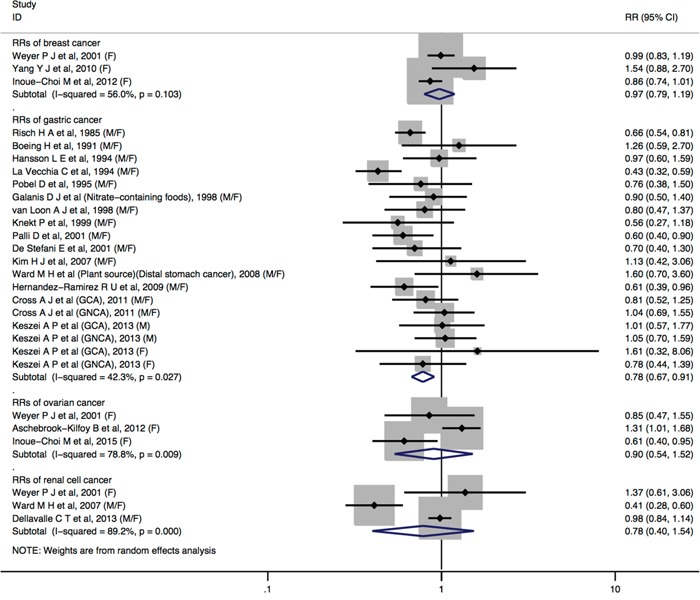
Forest plot (random-effects model) quantifying the relationships between dietary nitrate intake and breast cancer, gastric cancer, ovarian cancer, and renal cell cancer Squares indicate study-specific RRs (the size of the square reflects the study-specific statistical weight); horizontal lines indicate 95%CIs; the diamond indicates the summary RR estimate with its 95%CI. All statistical tests were two-sided. CI = confidence interval; RR = relative risk; M=Male; F=Female.

**Figure 3:**
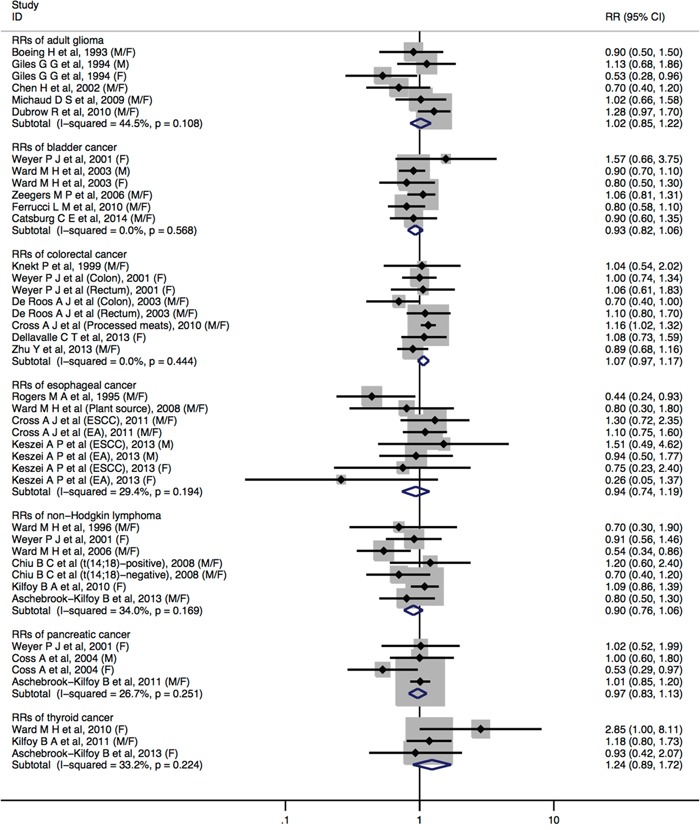
Forest plot (fixed-effects model) quantifying the relationships between dietary nitrate intake and adult glioma, bladder cancer, colorectal cancer, esophageal cancer, non-Hodgkin lymphoma, pancreatic cancer, and thyroid cancer Squares indicate study-specific RRs (the size of the square reflects the study-specific statistical weight); horizontal lines indicate 95%CIs; the diamond indicates the summary RR estimate with its 95%CI. All statistical tests were two-sided. CI = confidence interval; RR = relative risk; M=Male; F=Female.

Comparing the highest vs. the lowest levels of dietary nitrate intake, statistically significant inverse association was observed for gastric cancer (RR = 0.78, 95%CI = 0.67-0.91) (Figure 2). No significant associations were found between dietary nitrate intake and adult glioma (RR = 1.02, 95%CI = 0.85-1.22), breast cancer (RR = 0.97, 95%CI = 0.79-1.19), bladder cancer (RR = 0.93, 95%CI = 0.82-1.06), colorectal cancer (RR = 1.07, 95%CI = 0.97-1.17), esophageal cancer (RR = 0.94, 95%CI = 0.74-1.19), non-Hodgkin lymphoma (RR = 0.90, 95%CI = 0.76-1.06), ovarian cancer (RR = 0.90, 95%CI = 0.54-1.52), pancreatic cancer (RR = 0.97, 95%CI = 0.83-1.13), renal cell cancer (RR = 0.78, 95%CI = 0.40-1.54), and thyroid cancer (RR = 1.24, 95%CI = 0.89-1.72) (Figure 2 and Figure 3).

No statistically significant heterogeneity across studies was detected for dietary nitrate intake in relation to cancers of adult glioma, bladder cancer, colorectal cancer, esophageal cancer, non-Hodgkin lymphoma, pancreatic cancer, and thyroid cancer. We observed some heterogeneity for studies of dietary nitrate intake and breast cancer (*I*^2^ = 56.0%, *P*_heterogeneity_ = 0.103), gastric cancer (*I*^2^ = 42.3%, *P*_heterogeneity_ = 0.027), ovarian cancer (*I*^2^ = 78.8%, *P*_heterogeneity_ = 0.009), and renal cell cancer (*I*^2^ = 89.2%, *P*_heterogeneity_ <0.001).

In subgroup analyses of dietary nitrate intake and cancer risk of the colorectal, gastric and esophageal by sex, study design, study population, cancer type, most strata showed similar results, and there was no evidence of significant heterogeneity between-subgroups with meta-regression analyses (Table 2). When stratified by the adjustment for potential confounders, significant associations were observed among studies of gastric cancer without adjustment but not among studies with adjustment. The discrepancies are likely to be due to a small number of studies included in the analysis, especially about vitamin C intake.

**Table 2:** Summary relative risks (95% confidence intervals) of high vs. low levels of dietary nitrate intake in relation to cancer risk, stratified by selected study characteristics

Variable	Colorectal cancer				Gastric cancer				Esophageal cancer			
	Number of RRs	RR (95%CI)	P_h_^*^	P_h_^†^	Number of RRs	RR (95%CI)	P_h_^*^	P_h_^†^	Number of RRs	RR (95%CI)	P_h_^*^	P_h_^†^
Sex				0.820				0.167				0.783
Male	-	-	-		1	1.04(0.74-1.44)	0.913		1	1.05 (0.61-1.83)	0.471	
Female	1	1.08 (0.73-1.59)	-		1	0.85(0.49-1.46)	0.407		1	0.53 (0.20-1.37)	0.306	
Male and female	5	1.07 (0.97-1.18)	0.335		14	0.75(0.63-0.88)	0.030		3	0.95 (0.72-1.25)	0.080	
Study design				0.105				0.002				0.073
HC-CS	-	-	-		4	0.53(0.42-0.68)	0.115		-	-	-	
PC-CS	2	0.90 (0.74-1.10)	0.324		5	0.69(0.59-0.81)	0.144		2	0.55 (0.32-0.94)	0.297	
CS	4	1.12 (1.01-1.25)	0.915		6	0.92(0.78-1.09)	0.866		2	1.06 (0.82-1.39)	0.533	
Study populations				0.817				0.739				0.929
Asians	1	1.08 (0.73-1.59)	-		1	1.13(0.42-3.05)	-		-	-	-	
Americans	4	1.07 (0.97-1.18)	0.232		5	0.75(0.65-0.87)	0.120		3	0.95 (0.72-1.25)	0.080	
Europeans	1	1.04 (0.54-2.02)	-		8	0.77(0.61-0.98)	0.018		1	0.89 (0.55-1.43)	0.379	
Cancer subtypes				0.310				0.710				0.539
Colon/GCA/EA	2	0.90 (0.70-1.15)	0.200		2	0.90(0.64-1.27)	0.644		2	1.00 (0.73-1.38)	0.244	
Rectum/GNCA/ESCC	2	1.09 (0.80-1.48)	0.913		2	0.99(0.76-1.28)	0.672		2	1.22 (0.76-1.97)	0.656	
Adjustments in models												
Body mass index				0.578				0.780				0.854
No	4	0.99 (0.84-1.18)	0.735		11	0.74(0.65-0.86)	0.413		1	0.80 (0.30-1.80)	-	
Yes	2	1.10 (0.98-1.24)	0.080		4	0.78(0.59-1.04)	0.004		3	0.95 (0.74-1.21)	0.134	
Total energy intake				0.416				0.878				-
No	1	0.92 (0.69-1.23)	0.135		7	0.73(0.63-0.85)	0.357		-	-	-	
Yes	5	1.09 (0.98-1.20)	0.629		8	0.79(0.62-0.99)	0.011		4	0.94 (0.74-1.19)	0.194	
Cigarette smoking				0.567				0.022				
No	3	0.99 (0.83-1.18)	0.600		8	0.67(0.54-0.84)	0.050		-	-	-	-
Yes	3	1.10 (0.98-1.24)	0.213		7	0.91(0.77-1.07)	0.770		4	0.94 (0.74-1.19)	0.194	
Alcohol consumption				0.260				0.055				
No	5	1.10 (0.99-1.21)	0.565		9	0.68(0.55-0.85)	0.063		-	-	-	-
Yes	1	0.89 (0.68-1.16)	-		6	0.89(0.76-1.05)	0.575		4	0.94 (0.74-1.19)	0.194	
Vitamin C intake				0.820				0.336				0.854
No	5	1.07 (0.97-1.18)	0.335		13	0.76(0.65-0.89)	0.036		3	0.95 (0.74-1.21)	0.134	
Yes	1	1.08 (0.73-1.59)	-		2	0.98(0.63-1.54)	0.165		1	0.80 (0.30-1.80)	-	
Physical activity				0.412				0.061				0.073
No	4	1.10 (0.99-1.22)	0.437		13	0.72(0.60-0.86)	0.052		2	0.55 (0.32-0.94)	0.297	
Yes	2	0.95 (0.76-1.18)	0.422		2		0.872		2	1.06 (0.82-1.39)	0.533	
Family history of cancer				-				0.033				-
No	6	1.07 (0.97-1.17)	0.444		11	0.80(0.71-0.90)	0.340		4	0.94 (0.74-1.19)	0.194	
Yes	-	-	-		4	0.61(0.42-0.87)	0.087		-	-	-	

* *P* value for heterogeneity within each subgroup.

† *P* value for heterogeneity between subgroups with meta-regression analysis.

### Dietary nitrite intake and site-specific cancer risk

Twenty prospective and thirty-two case-control studies examined the association between dietary nitrite intake and cancer risk. Among all included studies, 19 studies were on gastric cancer, 7 studies on esophageal cancer, 6 studies each on colorectal cancer, adult glioma, 4 studies each on bladder cancer, non-Hodgkin lymphoma, 3 studies on breast cancer, 2 studies each on ovarian cancer, pancreatic cancer, renal cell cancer, thyroid cancer, and prostate cancer.

**Figure 4:**
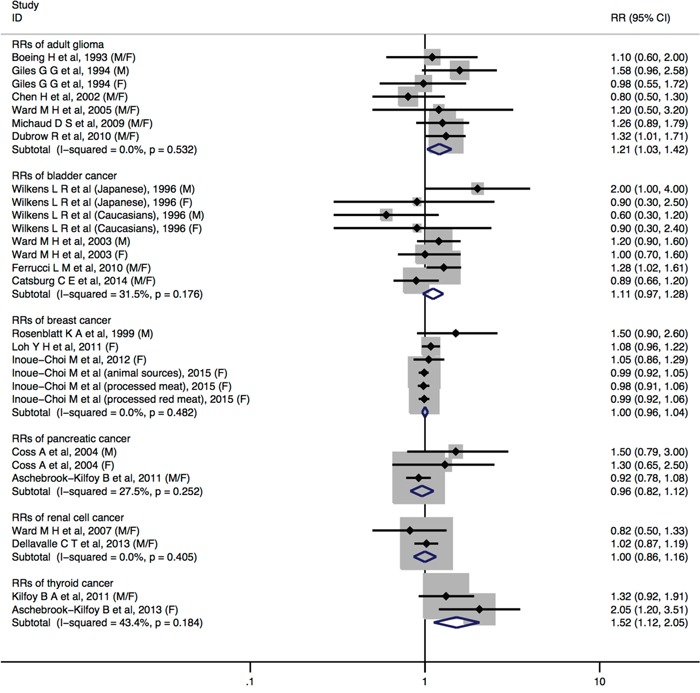
Forest plot (fixed-effects model) quantifying the relationships between dietary nitrite intake and adult glioma, bladder cancer, breast cancer, pancreatic cancer, renal cell cancer, and thyroid cancer Squares indicate study-specific RRs (the size of the square reflects the study-specific statistical weight); horizontal lines indicate 95%CIs; the diamond indicates the summary RR estimate with its 95%CI. All statistical tests were two-sided. CI = confidence interval; RR = relative risk; M=Male; F=Female.

**Figure 5:**
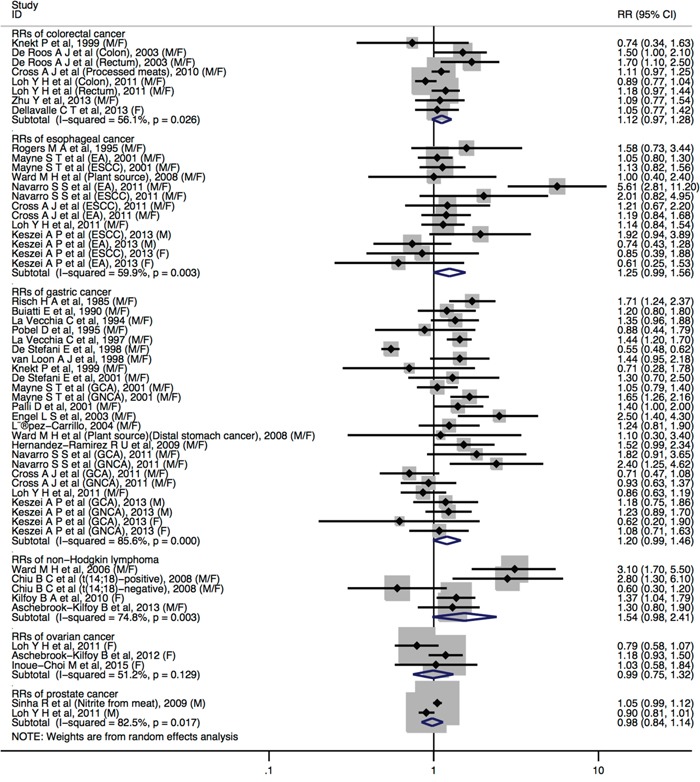
Forest plot (random-effects model) quantifying the relationships between dietary nitrite intake and colorectal cancer, esophageal cancer, gastric cancer, non-Hodgkin lymphoma, ovarian cancer and prostate cancer Squares indicate study-specific RRs (the size of the square reflects the study-specific statistical weight); horizontal lines indicate 95%CIs; the diamond indicates the summary RR estimate with its 95%CI. All statistical tests were two-sided. CI = confidence interval; RR = relative risk; M=Male; F=Female.

Individuals with highest nitrites consumption, compared with the lowest, increased the risk of adult glioma (RR= 1.21, 95%CI = 1.03-1.42), thyroid (RR= 1.52, 95%CI = 1.12-2.05) (Figure 4). No statistically significant associations were detected between dietary nitrite intake and bladder cancer (RR = 1.11, 95%CI = 0.97-1.28), breast cancer (RR = 1.09, 95%CI = 0.98-1.20), colorectal cancer (RR = 1.12, 95%CI = 0.97-1.28), esophageal cancer (RR = 1.24, 95%CI = 0.98-1.55), non-Hodgkin lymphoma (RR = 1.54, 95%CI = 0.98-2.41), ovarian cancer (RR = 0.98, 95%CI = 0.66-1.45), pancreatic cancer (RR = 0.96, 95%CI = 0.82-1.12), prostate cancer (RR = 0.98, 95%CI = 0.84-1.14), and renal cell cancer (RR = 1.00, 95%CI = 0.86-1.16). Furthermore, a borderline significant associations were found in gastric cancer (RR = 1.21, 95%CI = 0.99-1.47) (Figure 4 and Figure 5).

No statistically significant heterogeneity across studies was observed for dietary nitrite intake in relation to cancers of adult glioma, bladder, breast, ovarian cancer, pancreatic, renal cell, and thyroid. We observed heterogeneity for studies of dietary nitrite intake and colorectal cancer (*I*^2^ = 56.1%, *P*_heterogeneity_ = 0.026), esophageal cancer (*I*^2^ = 59.9%, *P*_heterogeneity_ = 0.003), gastric cancer (*I*^2^ = 85.6%, *P*_heterogeneity_ <0.001), non-Hodgkin lymphoma (*I*^2^ = 74.8%, *P*_heterogeneity_ = 0.003), ovarian cancer (I2 = 51.2%, P_heterogeneity_ = 0.129) and prostate cancer (*I*^2^ = 82.5%, *P*_heterogeneity_ = 0.017).

When stratified by subgroup of sex, study design, study population, cancer type of dietary nitrite intake in relation to cancers of the colorectal, gastric and esophageal, most strata showed similar results and no evidence of significant heterogeneity between-subgroups with meta-regression analyses (Table 3). In the adjustment for potential confounders analyses, there did not shown a significant difference between estimates adjusted and those not adjusted for specific factors in colorectal cancer and gastric cancer, except esophageal cancer. Although the deviation from adjustments of esophageal cancer most likely due to a small number of studies.

**Table 3:** Summary relative risks (95% confidence intervals) of high vs. low levels of dietary nitrite intake in relation to cancer risk, stratified by selected study characteristics

Variable	Colorectal cancer				Gastric cancer				Esophageal cancer			
	Number of RRs	RR (95%CI)	*P*_h_^*^	*P*_h_^†^	Number of RRs	RR (95%CI)	*P*_h_^*^	*P*_h_^†^	Number of RRs	RR (95%CI)	*P*_h_^*^	*P*_h_^†^
Sex				0.766				0.775				0.423
Male	-	-	-		1	1.21(0.93-1.58)	0.884		1	1.16 (0.46-2.95)	0.037	
Female	1	1.05 (0.77-1.42)	-		1	1.01(0.68-1.49)	0.365		1	0.74 (0.41-1.34)	0.588	
Male and female	5	1.13 (0.97-1.32)	0.014		18	1.23(0.99-1.54)	0.000		6	1.35 (1.04-1.75)	0.004	
Study design				0.092				0.616				0.214
HC-CS	-	-	-		5	1.07(0.65-1.74)	0.000		-	-	-	
PC-CS	2	1.37 (1.11-1.70)	0.227		8	1.52(1.34-1.73)	0.270		4	1.56 (0.99-2.45)	<0.001	
CS	4	1.04 (0.96-1.13)	0.114		5	1.01(0.88-1.17)	0.267		3	1.10 (0.92-1.32)	0.341	
Study populations				0.236				0.003				0.241
Asians/African	1	1.05 (0.77-1.42)	-		2	0.80(0.35-1.84)	0.009		-	-	-	
Americans	3	1.17 (1.05-1.31)	0.126		8	1.39(1.12-1.73)	0.003		5	1.41 (1.03-1.94)	0.002	
Europeans	2	0.99 (0.78-1.26)	0.065		9	1.25(1.13-1.38)	0.269		2	1.05 (0.83-1.31)	0.178	
Cancer subtypes				0.571				0.312				0.936
Colon/GCA/EA	2	1.12 (0.68-1.87)	0.011		4	1.04(0.84-1.28)	0.131		4	1.26 (0.74-2.14)	<0.001	
Rectum/GNCA/ESCC	2	1.35 (0.96-1.91)	0.116		4	1.32(1.01-1.73)	0.040		4	1.17 (0.93-1.46)	0.348	
Adjustments in models												
Body mass index				0.252				0.335				0.015
No	3	1.27 (1.04-1.55)	0.111		13	1.31(0.94-1.83)	0.000		2	2.32 (0.82-6.52)	0.009	
Yes	3	1.05 (0.96-1.14)	0.080		6	1.13(0.96-1.32)	0.030		5	1.09 (0.95-1.24)	0.554	
Total energy intake				0.042				0.571				-
No	1	1.59 (1.21-2.09)	-		6	1.35(0.80-2.27)	0.000		-	-	-	
Yes	5	1.04 (0.96-1.13)	0.186		13	1.19(1.07-1.31)	0.056		7	1.24 (0.98-1.55)	0.003	
Cigarette smoking				0.133				0.007				0.001
No	2	1.32 (1.08-1.62)	0.131		10	1.47(1.32-1.63)	0.371		1	3.50 (1.29-9.54)	-	
Yes	4	1.04 (0.96-1.13)	0.112		9	1.00(0.77-1.31)	0.000		6	1.09 (0.95-1.24)	0.644	
Alcohol consumption				0.318				0.016				0.001
No	4	1.16 (1.04-1.29)	0.121		11	1.45(1.30-1.61)	0.285		1	3.50 (1.29-9.54)	-	
Yes	2	1.03 (0.84-1.26)	0.073		8	1.03(0.78-1.35)	0.000		6	1.09 (0.95-1.24)	0.644	
Vitamin C intake				0.766				0.712				0.731
No	5	1.13 (0.97-1.32)	0.014		17	1.20(0.98-1.47)	0.000		6	1.25 (0.99-1.59)	0.002	
Yes	1	1.05 (0.77-1.42)	-		2	1.40(0.95-2.07)	0.681		1	1.00 (0.40-2.40)	-	
Physical activity				0.222				0.069				0.214
No	3	1.17 (1.04-1.31)	0.076		16	1.33(1.03-1.72)	0.000		4	1.56 (0.99-2.45)	<0.001	
Yes	3	1.00 (0.90-1.12)	0.149		3	0.98(0.84-1.14)	0.380		3	1.10 (0.92-1.32)	0.341	
Family history of cancer				-				0.447				-
No	6	1.12 (0.98-1.28)	0.026		16	1.18(0.95-1.47)	0.000		7	1.24 (0.98-1.55)	0.003	
Yes	-	-	-		3	1.39(1.13-1.71)	0.971		-	-	-	

* *P* value for heterogeneity within each subgroup.

† *P* value for heterogeneity between subgroups with meta-regression analysis.

### Dose-response analysis between dietary nitrate/nitrite intake and site-specific cancer risk

Limited number of studies available precluded any meaningful subgroup analyses for linear dose-response meta-analyses. Eighteen [6, 9, 15, 22, 23, 34, 35, 42, 43, 45, 46, 48, 50, 52–54, 61, 62] and twelve [5, 6, 9, 34, 42, 43, 45, 46, 48, 50, 52, 53] studies were included in the dose-response analysis for dietary nitrate and nitrite respectively. The summary RR for each increase by 10mg/day for dietary nitrate intake was 0.99 (95%CI = 0.98-1.00) with moderate to high heterogeneity (I2 = 63.4%). Increasing the dosage of dietary nitrite by 0.5mg/day, the pooled RR for cancer risk was 1.04 (95%CI = 0.99-1.08) with moderate heterogeneity (I2 = 70.6%) (data not shown). Publication bias was not detected by using Egger's test or Begg's test or by visual inspection of the funnel plot both for nitrate and nitrite intake.

### Publication bias and meta-regression

With regards to dietary nitrate intake and gastric cancer, funnel plot and Egger's regression test (P = 0.018) suggested publication bias, whereas Begg's rank correlation test (P = 0.208) did not. Adjusting the possible publication bias for nitrates using “trim and fill” method did not influence the pooled RR (RR = 0.65, 95%CI = 0.56-0.77). We did not evaluate publication bias for other cancer sites due to small numbers of studies for those sites.

No publication bias was evident for the relations of dietary nitrite intake to esophageal cancer (Begg's rank correlation test: *P* = 0.583; Egger's regression test: *P* = 0.349). A significantly publication bias was detected in gastric cancer by Egger's regression test (*P* = 0.024), whereas Begg's rank correlation test did not (*P* = 0.293). Adjusting the possible publication bias for gastric cancer using “trim and fill” method significantly influenced the conclusion (RR = 0.80, 95%CI = 0.65-0.99). To explore the possible heterogeneity between dietary nitrite and gastric cancer, we further conducted a meta-regression. We found that geographic area was the main source of heterogeneity for nitrites, which interpreted 73.83%(0.049/0.188) of the estimated between-study variance (τ^2^). We did not evaluate publication bias for other cancer sites due to small numbers of studies for those sites.

## DISCUSSION

Overall, the primary finding from our meta-analysis of epidemiologic studies indicated that consumption of food rich in nitrate was inversely associated with gastric cancer risk, but high intake of nitrite may result in an elevated cancer risk of adult glioma and thyroid.

Highest category of dietary nitrate intake had a 22% reduction in gastric cancer risk compared with that for the lowest intake category. We also found a weak increased association of gastric cancer risk among those who reported higher consumption of dietary nitrite. In addition, similar results were found in dose-response analyses between dietary nitrate/nitrite intake and cancer risk.

The association between dietary nitrate intake and cancer risk is tenuous. Some investigators observed an increase in gastric cancer risk with increasing nitrate consumption [49] whereas, others had observed no association [24, 25, 57, 59]; despite this, several biologic mechanisms may mediate the observed inverse association between dietary nitrate intake and gastric cancer risk. First, the reverse association for nitrate may be due to the protective effect of vegetables in the diet. The major sources of dietary nitrate are vegetables, which contain nutrients that inhibit the *in vivo* N-nitrosation in food, and its protection effect is likely to be reflected by vitamin C and other anti-oxidants [67]. For example, vitamin C and vitamin E have been shown to inhibit the formation of N-nitroso compounds from nitrate in human subjects [68]. Food components, such as vitamins C and E, may exert an inhibitory effect in cancer carcinogenesis by blocking the nitrosation process by quenching free radicals in their anaerobic reaction with nitrite, thus reducing the endogenous synthesis of NOCs [69]. Further studies with large sample size and sufficient statistical power are required to confirm or refute these findings.

In the present study, we observed higher consumption of food rich in nitrite probably increases the risk of adult glioma and thyroid cancer. However, it should be noted that only a few studies were included for each cancer in our meta-analysis. Associations between dietary nitrite intake and cancer risk were reported controversial in many previous studies. Most of the case-control studies [49, 70], but not all [57, 59], showed positive association between nitrite intake and gastric cancer risk, whereas three prospective studies did not support positively association [11, 13, 24]. These contradictory results may due to various sources of dietary nitrite. The main source of dietary nitrite is usually animal products (especially processed meats), which also contain amines and amides, precursors necessary for endogenous nitrosation [71]. As a result, dietary nitrite intake from animal products may result in more substantial exposure to NOCs than consumption from plant source products.

Our systematic review and meta-analysis have a number of important strengths. This meta-analysis included a large sample size of 60,627 cases and 4,730,572 non-cases, uniform criteria was applied for identifying relevant studies and abstracting pertinent information. In addition, our study considered a number of subgroups to explore heterogeneity. Still, consideration should also be given to potential limitations in this meta-analysis. First, this meta-analysis is based on observational studies, including case-control and cohort studies, and therefore, the potential study biases or residual confounder within the individual studies can affect the pooled estimate. Thus, larger studies, especially prospective studies, are warranted in the future. Second, nitrate and nitrite are potential human carcinogens under conditions preferable to endogenous nitrosation (e.g., vitamin C), but not many studies considered these factor; not many studies assessed nitrate and nitrite intake by specific food sources or stratified/adjusted for these factors. Concomitant consumption of dietary nitrite/nitrite and vitamin C is likely to be important for inhibition of endogenous nitrosation [72], but we were not able to assess this because of limited data source. Third, although food frequency questionnaire (FFQ) has been widely used to capture habitual dietary intake, the accuracy of FFQ remains a concern. Due to the lack of uniformity for exposure assessment across studies, measurement error in different studies was inevitable. However, we were unable to rule out variation in the FFQ, as most studies did not show FFQ accuracy and validation. Fourth, dietary nitrate and nitrite intake levels are different by country and region. There was a wide range of nitrates/nitrites intake values between the lowest and highest categories, and the present study including studies in different populations from multiple regions, which might lead to the heterogeneity in the pooled analysis. Nitrate contents in vegetables vary by fertilizer application practice, and nitrite contents in processed meats vary by food additive regulations. Nitrate intake is usually higher in Asian populations than in Western populations because Asian diets are more vegetable-based. Lastly, during the long follow-up for cohort studies, dietary intake level of nitrates, nitrites might have changed due to participants may have changed their dietetic patterns. Meanwhile, food-processing technology has developed as well. Further prospective studies with update dietary information are warranted.

In conclusion, findings from this meta-analysis provided modest evidence that dietary nitrate and nitrite intake were potentially associated with certain type of cancer risk. In the field of nutritional epidemiology, diet is a very complex and potentially modifiable exposure. Because of these limitations and confounding factors, we could not absolutely confirm the reliability of these findings. Future well-designed observational studies are warranted to further clarify the potential nitrate/nitrite and cancer association by subtypes and according to molecular classifications.

## MATERIALS AND METHODS

### Literature and search strategy

We identified studies through searching from database initiation until April 30^th^, 2016 using MEDLINE (PubMed; http://www.ncbi.nlm.nih.gov/pubmed) for both case-control and cohort studies that evaluated the association between dietary nitrate or nitrite intake and the risk of cancer. The search was limited to published studies in English and studies of humans by using the following keywords and Medical Subject Headings terms: (nitrate OR nitrite OR N-nitroso compounds) AND (cancer OR neoplasm OR carcinoma OR tumor). In addition, we carried out a manual retrieve of reference lists of included studies to identify other possible eligible articles that were not found in our primary search. We followed standard criteria for conducting and reporting meta-analyses. [73, 74]

### Study selection criteria

Published studies were included in the analysis based on the following criteria: (1) investigated the association between dietary nitrate and/or nitrite intake and cancer risk; (2) had a case-control or cohort study design; (3) provided odds ratio (OR), relative risk (RR), or hazard ratio (HR) estimates with its 95%CI or data necessary to calculate them. When multiple publications from the same study were available, we used the publication with the largest number of cases or the most-applicable information. To be eligible for dose-response analysis, the studies had to further provide quantitative measure of dietary nitrate/nitrite intake for at least three categories with the estimates of RRs, corresponding 95%CI, category-specific or total number of cases and category-specific or total number of either person-years or non-cases.

Finally, we identified 62 potentially relevant full-text publications from 3058 articles (Figure 1). In total, 49 publications reported dietary nitrate intake and cancer risk and 51 publications reported dietary nitrite intake and cancer risk. For the dose-response analyses, we included 18 publications for nitrate intake analysis and 12 publications for nitrite intake analysis.

### Data abstraction

Two investigators (L X and M M) independently performed the eligibility evaluation with the inclusion criteria and data abstraction. Disagreements were discussed and resolved by consensus or by involving a third reviewer (J Z). The following information was extracted from each eligible study by: (1) name of the first author; (2) year of publication; (3) origin of country; (4) study sample size (number of cases, and controls or cohort size); (5) gender; (6) duration of follow-up for cohort studies; (7) exposure and outcome assessment including dietary nitrate and/or nitrite intake category; (8) cancer type; (9) study-specific adjusted estimates with their 95%CIs for highest compared with lowest dietary nitrate/nitrite intake; (10) factors matched by or adjusted for in data analysis. If multiple estimates of the association were available, we abstracted the estimate that adjusted for most of confounders. If none were adjusted, we included the crude estimate. If no estimate was given, it was calculated with its corresponding 95%CI using raw data presented in the publication.

We did not adopt the Newcastle-Ottawa Scale [75] to assess the methodological quality of all included studies because quality scoring in a meta-analysis of observational studies is controversial, which might bring the analyst's subjective bias to the results and impedes the recognition of key sources of heterogeneity [76, 77]. Instead, we carried out some subgroups and sensitivity analyses.

### Statistical analysis

The study-specific adjusted RRs were used as common measure of association across studies. As absolute risk of cancer is relatively low, we assumed that estimates of ORs from case-control studies and risk, rate, or HRs from cohort studies were all valid estimates of the RR. Thus, we reported all results as the RR for simplicity [78]. The possible heterogeneity in results across studies was examined by using Cochran Q and quantified by *I^2^* statistics [79]. In Q statistic analysis, a *p*-value less than 0.1 were considered statistically significant of heterogeneity. When substantial heterogeneity was detected, the summary estimate based on the random-effects model (DerSimonian and Laird method) was reported [80]. Otherwise, the summary estimate based on the fixed effects model (the inverse variance method) was reported [81]. We used these two effects models to calculate summary RRs and 95%CI for the highest versus lowest categories of dietary nitrate/nitrite intake for the analysis. Also, heterogeneity between subgroups was evaluated by meta-regression. Subgroup analyses were carried out based on the cancer subsites, study design (cohort, hospital-based case-control and population-based case-control studies), geographic area (Europe, America, and Asia), gender (men vs. women). We also stratified the meta-analysis by potentially important confounders (ie, BMI, vitamin C intake, and smoking status).

In a further analysis, we pooled risk estimates related to nitrate/nitrite and risk of cancer according to 10mg/0.5mg per day of dietary intake. We used generalized least squares for trend estimation as described by Orsini et al [82]. In dose-response analyses, for each study, the midpoint of dietary nitrate/nitrite intake in each category was assigned to the corresponding RR. When the lowest category was open-ended, the lower boundary was set to 0. When the highest category was open-ended, the length of the open-ended interval was assumed to be the same as that of the adjacent interval. Publication bias was evaluated using Egger's linear regression [83] and Begg's rank correlation methods [84], and funnel plots. A *P* value of <0.05 for the two aforementioned tests was considered representative of significant statistical publication bias. All data analyses were carried out using Stata software (version 11.0; StataCorp, College Station, TX). *P* values were 2-sided with a significance level of 0.05.

## SUPPLEMENTARY MATERIALS TABLE




