# Supplementary material for: Association between dietary nitrate and nitrite intake and site-specific cancer risk: evidence from observational studies
Source: Oncotarget. 2016 Jul 29;7(35):56915–32. doi: 10.18632/oncotarget.10917 (PMC5302962; doi:10.18632/oncotarget.10917)
Supplement: Supplementary file 1 [file oncotarget-07-56915-s001.pdf]

Association between dietary nitrate and nitrite intake and site-specific cancer risk: evidence from observational studies

SUPPLEMENTARY TABLE

Supplementary Table S1: Characteristics of studies of dietary nitrate and nitrite intake and cancer risk*

See Supplementary File 1
