# Supplementary material for: Association between dietary nitrate and nitrite intake and site-specific cancer risk: evidence from observational studies
Source: Oncotarget. 2016 Jul 29;7(35):56915–32. doi: 10.18632/oncotarget.10917 (PMC5302962; doi:10.18632/oncotarget.10917)
Supplement: Supplementary file 2 [file oncotarget-07-56915-s002.docx]

**Supplementary table 1.** Characteristics of studies of dietary nitrate and nitrite intake and cancer risk *†

| **Cancer sites** | **First author (reference),**  **publication year, country, study design** | **Cases/subjects (age), gender, duration of follow-up** | **RR (95% CI) (highest vs. lowest level of dietary nitrate/nitrite intake)** | **Assessment of dietary nitrate/nitrite intake** | **Matched/adjusted factors** |
| --- | --- | --- | --- | --- | --- |
| Adult glioma | Michaud D S et al [1], 2009, United States, CS | 335/(Nurses’ Health Study I: 121,700 (30-55); Health Professionals Follow-Up Study: 51,529 (40-75); Nurses’ Health Study II: 116,686 (25-42), M/F, ≤24y | Nitrate (Q1 vs. Q5): RR = 1.02 (0.66, 1.58)  Nitrite (Q1 vs. Q5): RR = 1.26 (0.89, 1.79) | Self-administered FFQ | Age and caloric intake. |
| Adult glioma | Dubrow R et al [2], 2010, United States, CS | 585/545,770(50-71), M/F, 8y | Nitrate (Q1 vs. Q5): RR = 1.28 (0.97-1.70)  Nitrite (Q1 vs. Q5): RR = 1.32 (1.01-1.71) | Self-administered FFQ | Age, sex, race, energy intake, education, height , and history of cancer at baseline. |
| Adult glioma and meningioma | Boeing H et al  [3], 1993, Germany, PC-CS | 115 (glioma) and 81 (meningioma)/418 (25-75), M/F, NA | Nitrate (Q1 vs. Q3): Glioma OR = 0.9 (0.5-1.5)  Nitrite (Q1 vs. Q3): Glioma OR = 1.1 (0.6-2.0) | Standardized interview by trained interviewers | Age, gender, alcohol intake, and tobacco smoking. |
| Adult glioma | Giles G G et al  [4], 1994, Australia, PC-CS | 416/409 (20-70), M/F, NA | Nitrate (Q1 vs. Q3):  Males: OR = 1.13 (0.68-1.86)  Females: OR = 0.53 (0.28-0.96);  Nitrite (Q1 vs. Q3):  Males: OR = 1.58 (0.96-2.58)  Females: OR = 0.98 (0.55-1.72) | Self-administered questionnaire | Alcohol and tobacco. |
| Adult glioma | Chen H et al  [5], 2002, United States, PC-CS | 236/449 (≥21), M/F, NA | Nitrate (Q1 vs. Q4): OR = 0.7 (0.4-1.2)  Nitrite (Q1 vs. Q4): OR = 0.8 (0.5-1.3) | FFQ by telephone interview | Age, age-squared, gender, respondent type, education level, family history, and farming experience. |
| Adult glioma | Ward M H et al [6], 2005, United States, PC-CS | 251/498 (≥21), M/F, NA | Nitrite (<0.70 vs. ≥1.19mg/d):  OR = 1.2 (0.5-3.2) | FFQ by interviewers | Year of birth, gender, respondent type, ever live/work on a farm, education, beta-carotene, fiber, calories. |
| All cancer | Weyer P J et al [7], 2001, United States, CS | 3,150/21,977(55-69), F, 11y | Nitrate (<11.6 vs. >27.2mg):  All sites: RR = 0.98 (0.88-1.09)  Non-Hodgkin lymphoma: RR = 0.91 (0.56-1.46)  Leukemia: RR = 1.73 (1.00-3.00)  Colon: RR = 1.00 (0.74-1.34)  Rectum: RR = 1.06 (0.61-1.83)  Pancreas: RR = 1.02 (0.52-1.99)  Kidney: RR = 1.37 (0.61-3.06)  Bladder: RR = 1.57 (0.66-3.75)  Breast: RR = 0.99 (0.83-1.19)  Ovary: RR = 0.85 (0.47-1.55)  Uterine corpus: RR = 0.97 (0.68-1.39)  Lung and bronchus: RR = 0.78 (0.55-1.11)  Skin (melanoma): RR = 0.83 (0.43-1.61) | Semi-quantitative FFQ | Age and total energy. |
| All cancer | Loh Y H et al [8], 2011, United Kingdom, CS | 3,268/23,363 (40-79), M/F, 11.4y | Nitrite (Q1 vs. Q4):  All sites: RR = 1.02 (0.90-1.14)  Esophageal: RR = 1.14 (0.84-1.54)  Stomach: RR = 0.86 (0.63-1.19)  Colon: RR = 0.89 (0.77-1.04)  Rectum: RR = 1.18 (0.97-1.44)  Gastrointestinal: RR = 0.99 (0.89-1.10)  Breast: RR = 1.08 (0.96-1.22)  Prostate: RR = 0.90 (0.81-1.01)  Lung: RR = 0.97 (0.83-1.14)  Ovarian: RR = 0.79 (0.58-1.07) | Self-administered FFQ | Age, sex, BMI, cigarette smoking status, alcohol intake, energy intake, physical activity status, educational level, and menopausal status (in women). |
| Bladder cancer | Ferrucci L M et al [9], 2010, United States, CS | 854/300,933 (50-71), M/F, 7y | Nitrate (Q1 vs. Q5): RR = 0.80 (0.58-1.10)  Nitrite (Q1 vs. Q5): RR = 1.28 (1.02-1.61) | Self-administered FFQ | Age, sex, smoking, intakes of fruit, vegetables, beverages, and total energy |
| Bladder Cancer | Zeegers M P et al [10], 2006, Netherlands, CS | 889/120,852(55-69), M/F, 9.3y | Nitrate (57.4 vs. 158.9mg/day):  RR = 1.04 (0.80-1.36) | Self-administered FFQ | Age, sex, current smoking, smoking amount, smoking duration, and nitrate exposure from drinking water. |
| Bladder cancer | Wilkens L R et al [11], 1996, United States, PC-CS | 261/522 (NA), M/F, NA | Nitrite (Q1 vs. Q3):  Japanese Male: OR = 2.0 (1.0-4.0)  Japanese Female: OR = 0.9 (0.3-2.5)  Caucasians Male: OR = 0.6 (0.3-1.2)  Caucasians Female: OR = 0.9 (0.3-2.4) | Structured questionnaire by trained interviewer | Age, smoking status, pack-years, employment in a high-risk occupation, consumption of dark green vegetables in men and total vitamin C consumption in women. |
| Bladder cancer | Ward M H et al [12], 2003, United States, PC-CS | 808/1,259 (40-85), M/F, NA | Nitrate in men (<59 vs.≥119mg/d):  OR = 0.9 (0.7-1.1);  Nitrate in women (<62 vs.≥127mg/d):  OR = 0.8 (0.5-1.3);  Nitrite in men (<0.81 vs.≥1.39mg/d):  OR = 1.2 (0.9-1.6);  Nitrite in women (<0.58 vs.≥0.98mg/d): OR = 1.0 (0.7-1.6) | Self-administered FFQ | Age, cigarette smoking, education, duration of chlorinated surface water use study period. |
| Bladder cancer | Catsburg C E et al [13], 2014, United States, PC-CS | 1,660/1,586 (Mean: 54.4/54.4), M/F, NA | Nitrate (Q1 vs. Q5): OR = 0.90 (0.60-1.35)  Nitrite (Q1 vs. Q5): OR = 0.89 (0.66-1.20) | In-person structured interview | BMI, race/ethnicity, education, history of diabetes, total vegetable intake per day, vitamin A intake, vitamin C intake, carotenoid intake, total servings of food per day, smoking duration, and smoking intensity. |
| Breast cancer | Rosenblatt K A et al [14], 1999, United States, PC-CS | 220/291 (<65), M, NA | Nitrite (Q1 vs. Q4): OR = 1.5 (0.9-2.6) | Self-administered FFQ | Caloric intake. |
| Breast cancer | Yang Y J et al  [15], 2010, South Korea, HC-CS | 362/362(46.1±8.5), F, NA | Nitrate (Q1 vs. Q5): OR = 1.54 (0.88-2.70) | FFQ by trained interviewers | Multivitamin supplement, number of children, breast feeding, soy protein, and mushroom, and parity. |
| Breast cancer | Inoue-Choi M et al [16], 2012, United States, CS | 2,875/34,388 (61.6±4.2), F, 22y | Nitrate (Q1 vs. Q5): RR = 0.86 (0.74-1.01)  Nitrite (Q1 vs. Q5): RR = 1.05 (0.86-1.29) | Self-administered FFQ | Age, total energy intake, BMI, WHR, education, smoking, physical activity level, alcohol intake, family history of breast cancer, age at menopause, age at first live birth, estrogen use, total intake of folate, vitamin C and E and flavonoids, intakes of cruciferae and red meat. |
| Breast cancer | Inoue-Choi M et al [17], 2016, United States, CS | 9,305/193,742 (50-71), F, 9.4y | Nitrite (Q1 vs. Q5):  From animal sources: RR = 0.99 (0.92–1.05)  From processed meat: RR = 0.98 (0.91–1.06)  From processed red meat: RR = 0.99 (0.92–1.06) | Self-administered FFQ | Age, race, BMI, height, education level, cigarette smoking, alcohol intake, physical activity, familial history of breast cancer, age at menarche, age at menopause, age at first live birth, number of live births, hormone use, oral contraceptive use, numbers of previous breast biopsy, total calorie intake, total fat and fiber intake. |
| Colorectal cancer | Cross A J et al [18], 2010, United States, CS | 2,719/300,948 (50-71), M/F, 7y | Nitrate from processed meat (Q1 vs. Q5): RR = 1.16 (1.02-1.32)  Nitrite from processed meats (Q1 vs. Q5): RR = 1.11 (0.97-1.25). | Self-administered FFQ | Gender, education, BMI, smoking, intake of total energy, fiber, and dietary calcium. |
| Colorectal and other gastro-intestinal cancer | Knekt P et al [19], 1999, Finland, CS | 189/9,985 (15-99), M/F, 24y | Nitrate (Q1 vs. Q4):  Head and neck: RR = 0.84 (0.39-1.81)  Stomach: RR = 0.56 (0.27-1.18)  Colorectum: RR = 1.04 (0.54-2.02)  Nitrite (Q1 vs. Q4):  Head and neck: RR =0.83 (0.36-1.88)  Stomach: RR = 0.71 (0.28-1.78)  Colorectum: RR = 0.74 (0.34-1.63) | 1-year dietary history interview | Age, sex, municipality, smoking and energy intake. |
| Colorectal cancer | Dellavalle C T et al [20], 2014, China, CS | 619/73,118 (40-70), F, 11y | Nitrate (Q1 vs. Q5): RR = 1.08 (0.73-1.59)  Nitrite (Q1 vs. Q5): RR = 1.05 (0.77-1.42) | FFQ by trained interviewer | Age, energy intake, education, physical activity, dietary vitamin C intake, carotene and folate. |
| Colorectal cancer | De Roos A J et al [21], 2003, United States, PC-CS | 714/1,244 (40-85), M/F, NA | Nitrate (Q1 vs. Q4):  Colon cancer OR = 0.7 (0.4-1.0)  Rectum cancer OR = 1.1 (0.8-1.7)  Nitrite (Q1 vs. Q4):  Colon cancer OR = 1.5 (1.0-2.1)  Rectum cancer OR = 1.7 (1.1-2.5) | Questionnaire by mail | Age, sex, and estimates for rectum cancer are additionally adjusted for years served with chlorinated surface water. |
| Colorectal cancer | Zhu Y et al [22], 2014, Canada, PC-CS | 1,760/2,481 (20-74), M/F, NA | Nitrate (Q1 vs. Q5): OR = 0.89 (0.68-1.16)  Nitrite (Q1 vs. Q5): OR =1.09 (0.77-1.54) | FFQ by interview | Age, sex, energy intake, BMI, cigarette smoking status, alcohol consumption, physical activity, education attainment, household income, reported colon screening procedure, non-steroidal anti-inflammatory drug use, multivitamin supplement use, folate supplement use and province of residence. |
| Endometrial cancer | Barbone F et al  [23], 1993, United States, HC-CS | 168/334 (64), F, NA | Nitrate (Q1 vs. Q3): OR = 0.4 (0.2-0.8) | Semi-quantitative FFQ by interviewer | Age, race, years of schooling, total calories, use of unopposed estrogens, obesity, shape of obesity, smoking, age at menarche, age at menopause, number of pregnancies, diabetes, and hypertension. |
| Esophageal and Gastric Cancer | Mayne S T et al [24], 2001, United States, PC-CS | 1095/687 (30-79), M/F, NA | Nitrite (P25 vs. P75):  EA: OR = 1.05 (0.80-1.38)  ESCC: OR = 1.13 (0.82-1.56)  GCA: OR = 1.05 (0.79-1.40)  GNCA: OR = 1.65 (1.26-2.16) | FFQ by trained interviewer | Sex, site, age, race, proxy status, income, education, usual body mass index, cigarettes, years of consuming beer, wine, and liquor , and energy intake, sodium. |
| Esophageal and gastric cancer | Cross A J et al [25], 2011, United States, CS | 1,800/303,156(50-71), M/F, 10y | Nitrate (Q1 vs. Q5):  ESCC: RR = 1.30 (0.72-2.35)  EADC: RR = 1.10 (0.75-1.60)  GCA: RR = 0.81 (0.52-1.25)  GNCA: RR = 1.04 (0.69-1.55)  Nitrite (Q1 vs. Q5):  ESCC: RR = 1.21 (0.67-2.20)  EADC: RR = 1.19 (0.84-1.68)  GCA: RR = 0.71 (0.47-1.08)  GNCA: RR = 0.93 (0.63-1.37) | Self-administered FFQ | Age, sex, BMI, education, ethnicity, tobacco smoking, alcohol drinking, usual physical activity at work, vigorous physical activity, and the daily intake of fruit, vegetables, saturated fat, and calories. |
| Esophageal and Gastric Cancer | Navarro S S et al [26], 2011, United States, PC-CS | 282 (EA), 255 (GCA), 206 (ESCC), 352 (GNCA)/687 (30-79), M/F, NA | Meat/Nitrite(Food pattern) (Q1 vs. Q4):  EA OR = 5.61 (2.81-11.20)  ESCC OR = 2.01 (0.82-4.95)  GCA OR = 1.82 (0.91-3.65)  GNCA OR = 2.40 (1.25-4.62) | FFQ by trained interviewer | Gender, age, site, race, income, education, proxy status, energy intake, and mutual adjustment for other principle components. |
| Esophageal and gastric cancer | Keszei A P et al [27], 2013, Netherlands, CS | 924/120,852 (55-69), M/F, 16.3y | Nitrate (Q1 vs. Q3):  Male: ESCC: RR = 1.51 (0.49-4.62)  EA: RR = 0.94 (0.50-1.77)  GCA: RR = 1.01 (0.57-1.77)  GNCA: RR = 1.05 (0.70-1.59)  Female: ESCC: RR = 0.75 (0.23-2.40)  EA: RR = 0.26 (0.05-1.37)  GCA: RR = 1.61 (0.32-8.06)  GNCA: RR = 0.78 (0.44-1.39)  Nitrite (Q1 vs. Q3):  Male: ESCC: RR = 1.92 (0.94-3.89)  EA: RR = 0.74 (0.43-1.28)  GCA: RR = 1.18 (0.75-1.86)  GNCA: RR = 1.23 (0.89-1.70)  Female: ESCC: RR = 0.85 (0.39-1.88)  EA: RR = 0.61 (0.25-1.53)  GCA: RR = 0.62 (0.20-1.90)  GNCA: RR = 1.08 (0.71-1.63) | Self-administered questionnaire | Age, smoking status, years of cigarette smoking, number of cigarettes smoked per day, total energy intake, BMI, alcoholic intake, vegetable intake, fruit intake, level of education, and non-occupational physical activity. |
| Gastric cancer | Risch H A et al  [28], 1985, Canada, PC-CS | 565/429 (35-79), M/F, NA | Nitrate increase per 100g/day:  RR = 0.66 (0.54-0.81);  Nitrite increase per 1mg/day:  RR = 1.71 (1.24-2.37) | Interview | None. |
| Gastric cancer | Buiatti E et al [29], 1990, Italy, PC-CS | 1,016/1,159 (<75), M/F, NA | Nitrite (Q1 vs. Q5): OR = 1.2 (0.8-1.8) | Questionnaire by trained interviewers | Kilocalories and non-dietary variables. |
| Gastric cancer | Boeing H et al [30], 1991, Germany, HC-CS | 143/579 (<80), M/F, NA | Nitrate (Q1 vs. Q5): OR = 1.26 (0.59-2.70) | Questionnaire by trained interviewers | Age, sex, and hospital |
| Gastric cancer | La Vecchia C et al [31], 1994, Italy, HC-CS | 723/2,024 (61), M/F, NA | Nitrate (Q1 vs. Q5): OR = 0.43 (0.32-0.59)  Nitrite (Q1 vs. Q5): OR = 1.35 (0.96-1.88) | Standard questionnaire by trained interviewer | Age, sex, education, family history of gastric cancer, body mass index, and total energy intake. |
| Gastric cancer | Hansson L E et al [32], 1994, Sweden, PC-CS | 338/679 (40-79), M/F, NA | Nitrate (Q1 vs. Q4): OR = 0.97 (0.60-1.59) | FFQ by professional interviewers | Age, gender, ascorbic acid,β-carotene, α-tocopherol. |
| Gastric cancer | Pobel D et al [33], 1995, France, HC-CS | 92/128 (66.6±10.4), M/F, NA | Nitrate (Continuous variables):  OR = 0.76 (0.38-1.50)  Nitrite (Continuous variables):  OR = 0.88 (0.44-1.79) | Dietary history questionnaire by trained dietician | Age, sex, occupation and total calorie intake. |
| Gastric cancer | La Vecchia C et al [34], 1997, Italy, HC-CS | 746/2,053 (Median: 61/55), M/F, NA | Nitrite (<2.7 vs.≥2.7mg/d): OR =1.44 (1.2-1.7) | Self-administered questionnaire | Sex, age, and education. |
| Gastric cancer | De Stefani E et al [35], 1998, Uruguay, HC-CS | 340/698(25-84), M/F, NA | Nitrite (Continuous variables): OR = 0.55 (0.48-0.62) | FFQ by two trained social workers | Age, sex, residence, urban/rural status, smoking duration, alcohol consumption, and "mate" consumption. |
| Gastric cancer | Galanis D J et al [36], 1998, United States, CS | 108/11,907 (≥18), M/F, 14.8y | Nitrate-containing foods (0-3 vs. 8 or more times/week): RR = 0.9 (0.5-1.4) | Self-administered questionnaire | Age, years of education, Japanese place of birth, and gender (in combined analyses), cigarette smoking (for men), and alcohol intake status (for men). |
| Gastric cancer | van Loon A J et al [37], 1998, Netherlands, CS | 282/120,852 (55-69), M/F, 6.3y | Nitrate (55.8 vs. 172.2 mg/day):  RR = 0.80 (0.47-1.37)  Nitrite (0.01 vs. 0.35 mg/day):  RR = 1.44 (0.95-2.18) | Self-administered semi-quantitative FFQ | Age, sex, smoking, highest level of education, coffee consumption, intake of vitamin C and beta-carotene, family history of stomach cancer, prevalence of stomach disorders, use of refrigerator and use of freezer. |
| Gastric cancer | De Stefani E et al [38], 2001, Uruguay, HC-CS | 123/282 (30-89), M/F, NA | Nitrate (≤523 vs. >785mg/day): OR = 0.7( 0.4–1.3)  Nitrite (6.2 vs. >10.1mg/day): OR = 1.3( 0.7–2.5) | FFQ by trained social worker | Age, gender, residence, urban/rural status, tobacco smoking, alcohol drinking, mate drinking, total energy, protein,, total fat |
| Gastric cancer | Engel L S et al [39], 2003, United States, PC-CS | 368/695 (30-79), M/F, NA | Nitrite (Q1 vs. Q4):  OR = 2.50 (1.40-4.30) | In-person FFQ by trained interviewers | NA |
| Gastric cancer | López-Carrillo, 2004 [40], Mexico, HC-CS | 211/454 (>20), M/F, NA | Nitrite (0-0.11 vs. 0.27-2.25 portions/day):  OR = 1.24 (0.81-1.90) | Semiquantitative  Questionnaire by trained interviewers | Age, gender, residence, energy change in  socioeconomic level, years of education, Hp/CagA  status, and ascorbic acid |
| Gastric cancer | Kim H J et al  [41], 2007, Korea, HC-CS | 136/136(57.2 ± 13.9), M/F, NA | Nitrate (240 vs. 811 mg): OR = 1.13 (0.42-3.06) | FFQ by trained dietitians | Age, sex, socioeconomic status, family history, refrigerator use, H. pylori infection, and foods. |
| Gastric cancer | Palli D et al  [42], 2001, Italy, PC-CS | 382/562 (NA), M/F, NA | Nitrate (Q1 vs. Q3): OR = 0.6 (0.4-0.9)  Nitrite (Q1 vs. Q3): OR = 1.4 (1.0-2.0) | Self-administered FFQ | Age, sex, social class, family history of gastric cancer, area of rural residence, BMI. |
| Stomach and Esophagus | Ward M H et al [43], 2008, United States, PC-CS | 79/321 (≥21), M/F, NA | Nitrate from plant sources (<16.9 vs. >38.8mg/day):  Distal Stomach Cancer: OR = 1.6 (0.7-3.6)  Esophagus Cancer: OR = 0.8 (0.3-1.8)  Nitrite from plant sources (<0.36 vs. >0.67mg/day):  Distal Stomach Cancer: OR = 1.1 (0.3-3.4)  Esophagus Cancer: OR = 1.0 (0.4-2.4) | Health habits and history questionnaire with addition of foods high in nitrate and nitrite by interviewer | Year of birth, gender, education, smoking, alcohol, total calories, vitamin C, fiber, carbohydrate. |
| Gastric cancer | Hernandez-Ramirez R U et al [44], 2009, Mexico, PC-CS | 257/478 (≥20), M/F, NA | Nitrate (≤90.4 vs. >141.7mg/day):  OR = 0.61 (0.39-0.96);  Nitrite (≤1.0 vs. >1.2mg/day):  OR = 1.52 (0.99-2.34) | Interviewers administered FFQ | Energy, age, gender, H. pylori CagA status, schooling and consumptions of salt, chili and alcohol. |
| Hepatocellular carcinoma | Freedman N D et al [45], 2010, United States, CS | 338/495,006 (50-71), M/F, | Nitrate (Q1 vs. Q5): RR = 1.11(0.67-1.84)  Nitrite (Q1 vs. Q5): RR = 0.93(0.55-1.57) | Self-administered questionnaire | Age, sex, alcohol, body mass index, cigarette smoking, diabetes, education, fruit intake, vegetable intake, marital status , race and/or ethnicity, total energy from non-alcohol sources, usual physical activity throughout the day, and vigorous physical activity. |
| Laryngeal, esophageal, and oral cancer | Rogers M A et al [46], 1995, United States, PC-CS | 645/458 (NA), M/F, NA | Nitrate (<134 vs. >226mg):  Larynx: OR = 0.42 (0.22-0.80)  Esophagus: OR = 0.44 (0.24-0.93)  Oral cavity: OR = 0.46 (0.28-0.76)  Nitrite (<1.06 vs. >1.60mg):  Larynx: OR = 0.67 (0.34-1.34)  Esophagus: OR = 1.58 (0.73-3.44)  Oral cavity: OR = 0.66 (0.39-1.12) | FFQ by interviewers | Age, gender, pack-years of cigarettes, drink-years of alcohol, energy intake, ascorbic acid intake, body mass index, and level of education. |
| Nasopharyngeal carcinoma | Ward M H et al [47], 2000, Taiwan, HC-CS | 375/327(age 10, age 3 and weaning), M/F, NA | Nitrite from soybean products (Q1 vs. Q4):  Age 10: OR = 0.6 (0.3-1.4)  Age 3: OR = 1.2 (0.4-3.0)  Weaning: OR = 0.7 (0.3-1.4)  Nitrite from other foods (Q1 vs. Q4):  Age 10: OR = 2.0 (0.7-6.0)  Age 3: OR = 2.1 (0.4-10.2)  Weaning: OR = 2.8 (0.6-13.0) | Mothers were interviewed about children’s FFQ | Age, gender, ethnicity and vegetable intake. |
| Non-Hodgkin lymphoma | Ward M H et al [48], 1996, United States, PC-CS | 156/527 (≥21), M/F, NA | Nitrate (<13 vs. >26mg/d):  OR = 0.7 (0.3-1.9) | Standardized questionnaire by telephone interview | Age, gender, family history of cancer, vitamin C, and carotenes. |
| Non-Hodgkin lymphoma | Ward M H et al [49], 2006, United States, PC-CS | 458/383 (20-74), M/F, NA | Nitrate (<76 vs. >170mg/day):  OR = 0.54 (0.34-0.86)  Nitrite (<0.71 vs. 1.21mg/day):  OR = 3.1 (1.7-5.5) | Self-administered questionnaire | Age group, education, sex, study center, race, dietary vitamin C, and total energy. |
| Non-Hodgkin lymphoma | Chiu B C et al [50], 2008, United States, PC-CS | 147/1,075 (NA), M/F, NA | Nitrate in t(14;18)-positive group (<70 vs. >106 mg/day): RR = 1.2(0.6-2.4)  Nitrate in t(14;18)-negative group (<70 vs. >106 mg/day): RR = 0.7(0.4-1.2)  Nitrite in t(14;18)-positive group (<1 vs. >1 mg/day): RR = 2.8(1.3-6.1)  Nitrite in t(14;18)-negative group (<1 vs. >1 mg/day): RR = 0.6(0.3-1.2) | Questionnaire by telephone interview | Age, sex, type of respondent, family history of cancer, and body mass index. |
| Non-Hodgkin lymphoma | Kilfoy B A et al [51], 2010, United States, PC-CS | 594/710 (Mean:62.28/61.41), F, NA | Nitrate (Low vs. High):  OR = 1.09 (0.86-1.39)  Nitrite (Low vs. High):  OR = 1.37 (1.04-1.79) | Self-administered semi-quantitative FFQ by mail | Age, family history of cancer, calories, vitamin C intake, vitamin E intake, and protein intake. |
| Non-Hodgkin lymphoma | Aschebrook-Kilfoy B et al [52], 2013, United States, PC-CS | 348/470 (20-75), M/F, NA | Nitrate (Q1 vs. Q4): OR = 0.8 (0.5-1.3)  Nitrite (Q1 vs. Q4): OR = 1.3 (0.8-1.9) | Self-administered FFQ | Age, sex, body mass index, education, family history of cancer, vitamin C, and total daily caloric intake. |
| Ovarian cancer | Aschebrook-Kilfoy B et al [53], 2012, United States, CS | 709/151,316 (50-71), F, 10y | Nitrate (Q1 vs. Q5): RR = 1.31 (1.01-1.68)  Nitrite (Q1 vs. Q5): RR = 1.18 (0.93-1.50) | Self-administered FFQ | Age, race, total energy intake, family history of cancer, body mass index, education , smoking menopausal status, parity, age at menarche, and total daily dietary vitamin C intake. |
| Ovarian cancer | Inoue-Choi M et al, 2015 [54], United States, CS | 315/28,555 (55-69), F, 12y | Nitrate (Q1: 3.87-65.43 vs. Q5: 165.54-2,083.52 mg/day): RR = 0.61 (0.40–0.95)  Nitrite (Q1:0.11-0.80 vs. Q5:1.537-7.13 mg/day): RR = 1.03 (0.58–1.84) | Self-administered FFQ | Age, BMI, family history of ovarian cancer, number of live births, age at menarche, age at menopause, age at first live birth, oral contraceptive use, estrogen use, history of unilateral oophorectomy and total energy intake, cruciferous vegetable and red meat intake. |
| Pancreatic Cancer | Aschebrook-Kilfoy B et al [55], 2011, United States, CS | 1,728/303,156(50-71), M/F, 10y | Nitrate (Q1 vs. Q5): RR = 1.01 (0.85-1.20)  Nitrite (Q1 vs. Q5): RR = 0.92 (0.78-1.08) | Self-administered FFQ | Age, race, total energy intake, smoking status, family history of cancer, family history of diabetes, body mass index, and intakes of saturated fat, folate, and vitamin C. |
| Pancreatic Cancer | Coss A et al [56], 2004, United States, PC-CS | 189/1,244 (40-85), M/F, NA | Nitrate (<58 vs. >117mg/day):  Male OR = 1.00 (0.60-1.80)  Female OR = 0.53 (0.29-0.97)  Nitrite (<0.75 vs. >1.30mg/day):  Male OR = 1.50 (0.79-3.00)  Female OR = 1.30 (0.65-2.50) | Questionnaire by trained interviewers | Age, cigarette use, and caloric intake. |
| Prostate cancer | Sinha R et al [57], 2009, United States, CS | 10,313/175,343 (50-71), M, 9y | Nitrate from meat (Q1 vs. Q5):  RR = 1.06 (0.99-1.13)  Nitrite from meat (Q1 vs. Q5):  RR = 1.05 (0.99-1.12) | Self-administered FFQ | Age, total energy intake, race/ethnicity, education, marital status, family history of prostate cancer, undergoing prostate-specific antigen testing in the past 3 years, history of diabetes, body mass index, smoking history, frequency of vigorous physical activity, and intakes of alcohol, calcium, tomatoes, α-linolenic acid, vitamin E , zinc, and selenium. |
| Renal cell cancer | Dellavalle C T et al [20], 2013, United States, CS | 1,816/491,841 (50-71), M/F, 9y | Nitrate (2.09-24.90 vs.70.94-864.63 g/1000kcal): RR = 0.98 (0.84-1.14)  Nitrite (0.01-0.52 vs. 0.82-4.00g/1000kcal): RR = 1.02 (0.87-1.19) | Self-administered FFQ | Age, sex, caloric intake, race, smoking status, family history of cancer, BMI, alcohol intake, education, history of hypertension, history of diabetes. |
| Renal cell cancer | Ward M H et al [58], 2007, United States, PC-CS | 201/1,244 (40-85), M/F, NA | Nitrate (<53.92 vs. ≥122.01mg/d):  OR = 0.41 ((0.28-0.60));  Nitrite (<0.70 vs. 1.26mg/d):  OR = 0.82 ((0.50-1.33)) | Self-administered FFQ | Age, gender, sodium, total fat, total calories |
| Thyroid cancer | Ward M H et al [59], 2010, United States, CS | 45/21,977(55-69), F, 19y | Nitrate (≤17.4 vs. >41.1mg/day):  RR = 2.85 (1.00-8.11) | Self-administered semi-quantitative FFQ | Age, total calories, vitamin C intake, and residence location. |
| Thyroid cancer | Kilfoy B A et al [60], 2011, United States,  CS | 307/490,194 (50-71), M/F, 7y | Nitrate (Q1 vs. Q5): RR = 1.18 (0.80-1.73)  Nitrite (Q1 vs. Q5): RR = 1.32 (0.92-1.91) | Self-administered FFQ | Entry age, sex (overall model), smoking status, calories, race, family history, education, BMI, physical activity, alcohol use, vitamin C, beta-carotene, and folate. |
| Thyroid cancer | Aschebrook-Kilfoy B et al [61], 2013, China, CS | 164/73,317 (40-70), F, 11y | Nitrate (Q1 vs. Q4): RR = 0.93(0.42-2.07)  Nitrite (Q1 vs. Q4): RR = 2.05 (1.20-3.51) | FFQ by investigators | Age, total energy intake, education, history of thyroid disease, vitamin C, carotene, and folate intake. |

* CS = cohort study; HC-CS = hospital-based case-control study; PC-CS = population-based case-control study; M = male; F = female; FFQ = Food frequency questionnaire; EA = esophageal adenocarcinoma; ESCC = esophageal squamous cell carcinoma; GCA = gastric cardia adenocarcinoma; GNCA = gastric noncardia adenocarcinoma; RR = relative risk; OR = Odds ratio; CI = confidence interval; NA = not available.

† In-text citation for Supplementary table 1 is corresponding to the reference of main manuscript.
